# Relationship between transfusion of packed red blood cells, plasma free hemoglobin and storage time in patients with severe trauma

**DOI:** 10.1186/2197-425X-3-S1-A916

**Published:** 2015-10-01

**Authors:** S Chacón Alves, M Chico Fernández, C García Fuentes, A Del Pino Ramírez, N Zurera Plaza, L Umezawa Makikado, E Alted López, JC Montejo González

**Affiliations:** Hospital Universitario Doce de Octubre, Medicina Intensiva, UCI de Trauma y Emergencias, Madrid, Spain

## Introduction

Transfusion is a treatment in continuing debate and controversy. In recent years, there has been an increased interest about the "storage lesions" and its possible clinical consequences, and in addition, there has been an increased concern about the effect of the "age" of packed red blood cells.

## Objectives

Determining the influence of the age of the packed cells in stable trauma patients in hemolysis parameters and in the performance of transfusion.

## Methods

Prospective, descriptive, observational study on a cohort of patients with severe trauma, that were admitted more than 72 hours in the ICU of Trauma and Emergency of a high complexity hospital, without active bleeding. November 2012 to January 2014. It was measured hemoglobin (hb), hematocrit (hct), plasma free hemoglobin (fhb) by HemoCue^®^, bilirrubin and LDH before and after transfusion of one packed red blood cells. The attending physician determined the transfusion requirement, individually. We collected data about storage and characteristics of packed red blood cells and their age ("young blood" if it was less than 14 days and "old blood" if it was more than 14 days). Statistical data were analyzed by SPSS 16.0 considering statistically significant at P < 0.05.

## Results

34 transfusions were performed, with average threshold of 6.8 ± 0.3 g / dl for hb and 20.3% ± 1.6% for hematocrit. The most common blood group was the group O+ (56% of transfusions). The plasma free hemoglobin average pre-transfusion was 0.13 ± 0.06 g/dl compared to 0.19 ± 0.06 g/dl post-transfusion (p < 0.05). Increasing bilirrubin level was significant too, with a mean value of pre-transfusion 0.88 ± 0.90 mg/dl vs 1.14 ± 1.17 mg/dl post-transfusion. The average pre-transfusion LDH was 371.24 ± 197.75 U/L and posttransfusion was 396.39 ± 199.46 U/L (p = 0.06). These figures vary depending on the age of the blood, with an average increase fhb of 0.057 ± 0.11 g/dl for young blood versus 0.064 ± 0.062 g/dl for old blood (p > 0,05)

Hb performance adjusted to body surface is higher in young versus old blood, with an average of 0.91g / dl per kg / m2 ± 0.58 vs 0,51g / dl per kg / m2 ± 0.13 for hb and 2.88% per Kg / m2 ± 1.84 vs 1.57% per Kg / m2 ± 0.52 for hematocrit (p < 0.001).

## Conclusions

The transfusion of one packed red blood cells increases BPH and hemolysis significantly. Young blood transfusion has higher performance.
